# Imaging Tissue Physiology In Vivo by Use of Metal Ion-Responsive MRI Contrast Agents

**DOI:** 10.3390/ph13100268

**Published:** 2020-09-24

**Authors:** Pooyan Khalighinejad, Daniel Parrott, A. Dean Sherry

**Affiliations:** 1 Advanced Imaging Research Center, University of Texas Southwestern Medical Center, Dallas, TX 75390, USA; pooyan.khalighinejad@utsouthwestern.edu; 2 Department of Radiology, University of Texas Southwestern Medical Center, Dallas, TX 75390, USA; daniel.parrott@utsouthwestern.edu; 3 Department of Chemistry & Biochemistry, University of Texas at Dallas, Richardson, TX 75080, USA

**Keywords:** metal ion-responsive MRI agents, zinc secretion, macrocyclic chemistry, gadolinium-based contrast media

## Abstract

Paramagnetic metal ion complexes, mostly based on gadolinium (Gd^3+^), have been used for over 30 years as magnetic resonance imaging (MRI) contrast agents. Gd^3+^-based contrast agents have a strong influence on T_1_ relaxation times and are consequently the most commonly used agents in both the clinical and research environments. Zinc is an essential element involved with over 3000 different cellular proteins, and disturbances in tissue levels of zinc have been linked to a wide range of pathologies, including Alzheimer’s disease, prostate cancer, and diabetes mellitus. MR contrast agents that respond to the presence of Zn^2+^ in vivo offer the possibility of imaging changes in Zn^2+^ levels in real-time with the superior spatial resolution offered by MRI. Such responsive agents, often referred to as smart agents, are typically composed of a paramagnetic metal ion with a ligand encapsulating it and one or more chelating units that selectively bind with the analyte of interest. Translation of these agents into clinical radiology is the next goal. In this review, we discuss Gd^3+^-based MR contrast agents that respond to a change in local Zn^2+^ concentration.

## 1. Introduction

Magnetic resonance imaging (MRI) is now widely accepted as one of the most important diagnostic imaging tools available to clinicians because it is non-invasive, widely available, and provides anatomical images with high spatial and temporal resolution [1]. Since its first introduction into clinical medicine in the 1970s, MRI has improved significantly, mostly due to improvements in radio-frequency transmitters and receivers, gradient systems, faster computers, and post-processing software [2]. While MRI was initially used only as a tool for anatomical display, more recently, its role in imaging functional properties is extending and evolving, and the use of various contrast agents has been a critical component of that history [3].

Paramagnetic metal ion complexes, mostly based on gadolinium, have been used as MRI contrast agents since the first agent appeared on the market in 1986 [4]. The different signal intensities seen as grayscale intensity differences in MR images reflect differences in water and fat content in various tissues and the relaxation characteristics of those water, fat, and other protons, specifically their T_1_ and T_2_ relaxation times [1]. The influence of paramagnetic complexes on water proton relaxation can be rather dramatic depending upon the concentration of the agent. Even a small decrease in T_1_ and/or T_2_ can change the appearance of an image rather dramatically by brightening or darkening the water intensity in those tissues where the agent is present. Probably the most widely used application of contrast agents is for dynamic contrast enhancement (DCE) studies of tissue perfusion. This method alone has been useful in the evaluation of many malignancies, inflammations, infections, and vascular abnormalities [5]. Gadolinium (Gd^3+^)-based contrast agents have a strong influence on T_1_ relaxation times and are consequently the most commonly used agents in both the clinical and research environments.

In 1997, a new concept of a “smart” contrast agent that “turns on” or “turns off” only under specific conditions was introduced to the research world by Moats et al. [6]. In that first high-impact publication, Moats et al. demonstrated the simple concept of enzymatic cleavage of a galactose unit covalently bound to a Gd^3+^ complex in a position normally occupied by an exchanging water molecule. After cleaving the galactose unit from the complex, this allowed greater access of water molecules to the inner-sphere of the Gd^3+^ ion, a shortening of T_1_, and a corresponding increase in water proton intensity in an image. In principle, this could be used to image gene expression by visualizing β-galactosidase enzyme activity by MRI, providing that enzymatic cleavage of the galactose unit is fast compared to clearance of the agent. That single paper had a dramatic influence on the thinking of other scientists in subsequent years, and multiple contrast agents have since been reported to have specific triggers that alter either T_1_ or T_2_, including metal cations such as divalent Zn^2+^ [7]. Since many disorders have been linked to abnormal homeostasis of Zn^2+^, including diabetes, prostate cancer, and Alzheimer’s disease, MR detection of Zn^2+^ homeostasis potentially has multiple clinical indications. In this review, we will discuss and show applications of Gd^3+^-based MR contrast agents that respond to a change in local Zn^2+^ concentration.

## 2. Detection of Zinc with Magnetic Resonance Imaging

### 2.1. The Role of Zinc in Human Physiology

Zinc is the second most abundant metal in the human body and is essential for a variety of biological processes [8]. Divalent zinc (Zn^2+^) plays a structural and/or catalytic role in about 3000 human proteins, corresponding to ~10% of the human proteome [9]. Therefore, many cellular processes depend upon cellular Zn^2+^, including DNA transcription, protein synthesis, intercellular signaling, and intracellular signaling [10]. One of the most important functions of Zn^2+^ is its role in redox modulation, a key to the proper functioning of the immune system [11]. Given that Zn^2+^ has only one stable oxidation state in aqueous solution, its connection to redox is certainly not obvious, but this example illustrates the impact a single form of ionic Zn^2+^ can have on many biological systems, even though some of these effects, such as redox, are indirect effects. Zn^2+^-related metabolism has been linked to the development of many disorders including diabetes [12], prostate cancer [13], benign prostatic hyperplasia (BPH) [14], Alzheimer’s disease [15], multiple sclerosis [16], epilepsy [17], leukemia [18], other cancers [19], and more recently, COVID-19 [20,21].

Thus, tissue levels of available Zn^2+^ must be tightly regulated. This is exemplified by the relatively tight range of “free” Zn^2+^ ion concentrations in plasma, 12–16 μM. Here, “free” Zn^2+^ refers to ions weakly associated with anions and proteins in plasma, perhaps better defined as Zn^2+^ that is readily available for binding with small-molecule chelating ligands such as EDTA [22]. The distribution of Zn^2+^ in tissues and individual cells is controlled by 24 different membrane transporters, multiple metallothioneins (MTs), a zinc-specific transcription factor, and other mechanisms [23]. In comparison, homeostasis of iron, the most common metal ion in the human body, is controlled largely by only one importer and one exporter protein [8].

Although many roles of Zn^2+^ have been identified in cellular processes, our understanding of the specific mechanism of Zn^2+^ control in cellular physiology and distribution is limited. One important reason for this limitation is the inadequate available techniques for tracking Zn^2+^ in biological systems [24]. The development of fluorescent probes has provided many new insights into Zn^2+^ biology using optical techniques, but these have been largely limited to cell studies [25,26]. MRI has a lower sensitivity compared to optical probes but provides the opportunity to image at least extracellular Zn^2+^ levels nearly anywhere in deep tissues. Recently, multiple MR contrast agents have been developed to detect zinc, and these are discussed later in this review. We anticipate that when and if MR zinc sensors reach the clinical stage, the best target organs for detecting Zn^2+^ would be those with the most abundant zinc, including the pancreas (both acinar and β-cells) [27], prostate (especially in the peripheral zone) [28], and brain (especially in the hippocampus and neurodegenerative plaques) [29].

### 2.2. Contrast Agents in Magnetic Resonance Imaging

Paramagnetic contrast agents alter the appearance of an image typically by shortening the T_1_ or T_2_ of water protons. Although Gd^3+^-based agents are typically referred to T_1_ agents because they have the biggest impact on the T_1_ of water protons, they certainly impact both T_1_ and T_2_ [1]. Although complexes of other paramagnetic ions, including Mn^2+^, Mn^3+^, Fe^3+^, and Cu^2+^, have been proposed as MRI contrast agents, they each impact water proton relaxation and hence image contrast through somewhat different mechanisms. There are other categories of contrast agents also available or under development, including super-paramagnetic iron oxide (SPIO) particles and various other classes of nanoparticles [30], but we will focus on the design of agents that “respond” to the presence of Zn^2+^ ions in the remainder of this review.

**Figure 1:**
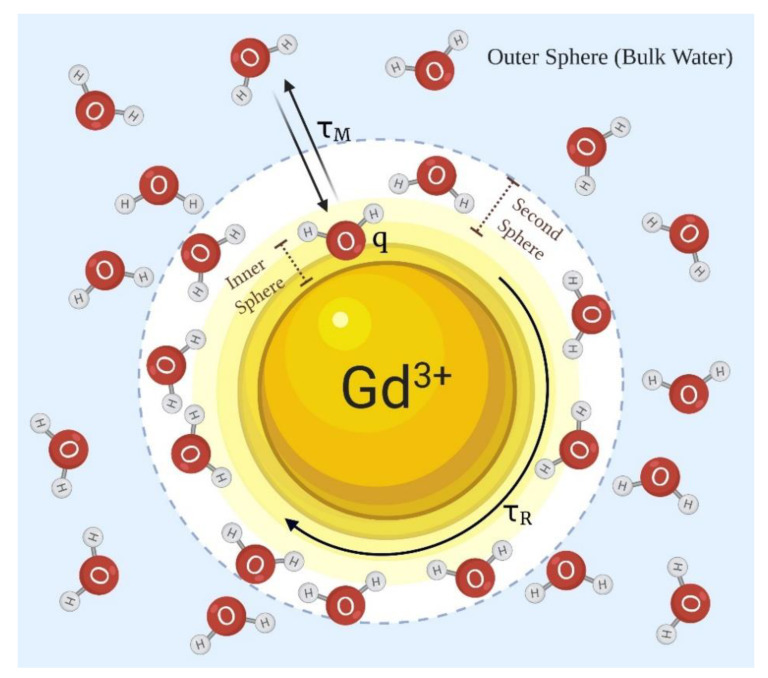
The variables that contribute most to the relaxation efficiency (r_1_) of a Gd^3+^-based contrast agent include the hydration number (q), the mean residence lifetime of the inner-sphere water molecule (τ_m_), and the rotational correlation time (τ_R_) of the entire Gd^3+^-complex.

Solvent water molecules in the presence of a Gd^3+^-based contrast agent can be described at a minimum as three types (Figure 1): (1) typically, a single inner-sphere water molecule that binds weakly with the Gd^3+^ ion and exchanges rather rapidly with all other nearby water molecules, (2) a number of second-sphere water molecules that interact weakly with the entire Gd^3+^-complex, and (3) the remaining outer-sphere or bulk water molecules [31]. All protons on all water molecules are influenced by the presence of a Gd^3+^ complex in solution, the largest impact is felt by the single exchanging inner-sphere water molecule. This single water molecule, closest to the paramagnetic center, is quickly relaxed by the seven unpaired electrons on the Gd^3+^ and then dissociates from the inner-sphere position and is replaced by another nearby water molecule. The partially oriented water molecules in the second-sphere are also partially relaxed by the nearby Gd^3+^ ion, but they are further away from the paramagnetic center, so the relaxation efficiency is about 50% less than the single inner-sphere water molecule. If water exchange is rapid, then the entire sample of water molecules is impacted by the presence of Gd^3+^, and this relaxation effect translates into a brighter signal intensity in T_1_-weighted proton images [32].

The efficiency of a MRI contrast agent is reported as a ratio of water proton relaxation rate (1/T_1_) per unit concentration (in mM), and given the symbols r_1_ for longitudinal relaxivity and r_2_ for transverse relaxivity. The longitudinal relaxivity (r_1_) is governed by the sum of inner-sphere relaxivity (r_1_^IS^) plus outer-sphere relaxivity (r_1_^OS^). The impact of q and τ_m_ on the relaxivity can be quantified using the following equations, in which [CA] is the concentration of the contrast agent in units of mM, P_m_ is the mole fraction of the bound water nuclei, and T_1m_ is the relaxation enhancement of the inner-sphere water [31,33,34,35]:

(1) r1IS=1T1IS[CA]

(2) $$\frac{1}{T_{1}^{\mathrm{IS}}}=\frac{{\mathrm{qP}}_{m}}{T_{1m}+\tau_{m}}$$

The impact of τ_R_ on r_1_ is more complex yet reasonably well-described by Solomon, Bloembergen, and Morgan’s theory [34,35], a theory that also includes the overall magnetic moment of the complex generated by seven unpaired electrons on the Gd^3+^. In overly simplified terms, when the molecular weight of the Gd^3+^ complex is relatively low, the complex rotates rather quickly and randomly in solution, so the impact of the seven unpaired electrons tends to average out such that the magnetic moment “feels” smaller to the exchanging water protons. However, when a Gd^3+^ complex interacts with a larger molecular structure, such as the human serum albumin (HSA), molecular rotation slows, and the water protons experience, on average, a larger effective magnetic moment generated by those same seven unpaired electrons. This can result in a substantially shorter water proton T_1_, an increase in r_1_, and a more intense water signal in a T_1_-weighted MR image [36,37]. This serves as the basis of how many “responsive” MRI contrast agents work.

### 2.3. “Responsive” MRI Contrast Agents

Clinically available Gd^3+^-based contrast agents are referred to as extracellular agents because they distribute into all tissue extracellular space and have little tissue specificity. Most are cleared largely via renal filtration, but a few agents having one or more organic side-chains display more liver uptake [38]. None are known to enter cells to any significant extent and none are known to experience a change in r_1_ in response to some physiological event, such as a change in pH, O_2_ concentration, temperature, or other biological events.

#### 2.3.1. Zinc-Responsive Contrast Agents

**Figure 2:**
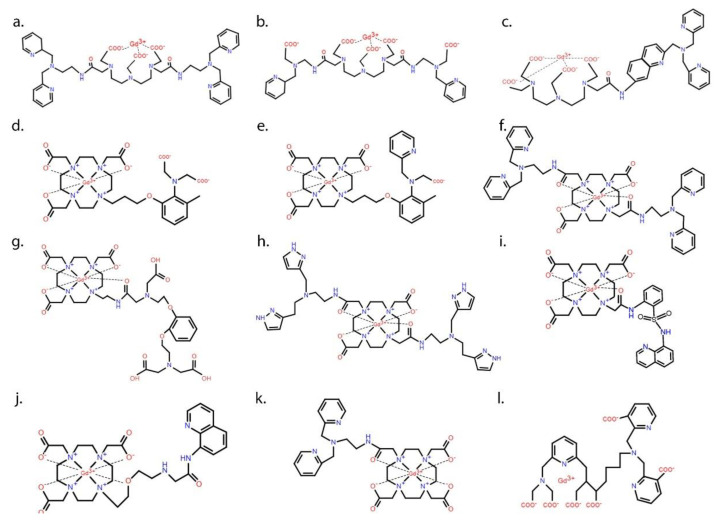
Chemical structures of some of the zinc-responsive gadolinium-based MR contrast agents. A common structural motif of most agents is a high-affinity chelating group such as DTPA (structures **a**–**c**) or DOTA (structures **d**–**l**) for chelating Gd^3+^ plus one or two appended lower affinity recognition sites for binding with Zn^2+^. The basic design of each agent was to trigger a change in r_1_ of Gd^3+^ upon binding of Zn^2+^ in its recognition site by either a change in hydration number (q), the mean residence lifetime of the inner-sphere water molecule (τ_m_), or the rotational correlation time (τ_R_) of the entire Gd^3+^ complex.

Zn^2+^-responsive MR contrast agents are typically composed of a paramagnetic metal ion encapsulated by a high-affinity ligand such as DTPA or DOTA plus one or more weaker chelating side-chain groups to serve as Zn^2+^ recognition binding sites. The structures and properties of several Gd^3+^-based Zn^2+^ sensors reported in the literature are shown in Figure 2 and summarized in Table 1, respectively.

In 2001, Hanaoka et al. reported the first zinc-responsive MR contrast agent (GdL^c^, Figure 2a), a Gd^3+^-DTPA derivative with two BPEN (N,N-bis (2-pyridyl-methyl) ethylenediamine) side chains for binding with Zn^2+^ [39]. It was likely disappointing to find that the r_1_ of this agent decreased after addition of the first Zn^2+^ ion (see Table 1) and remained low after addition of a second equivalent of Zn^2+^. These authors concluded that the first Zn^2+^ ion formed a strong tetrahedral complex with the four pyridine ligands from the two BPEN moieties and this restricted access of water to the inner-sphere of the Gd^3+^ ion. Although this first agent did not prove suitable for in vivo imaging of Zn^2+^, the idea of using BPEN for Zn^2+^ recognition because of the high selectivity of the dipicolylamine (DPA) groups for Zn^2+^ over other common biological cations such as Ca^2+^, Mg^2+^, K^+^, and Na^+^ did find use in later successful designs (Figure 2). However, it should be pointed out that BPEN shows little to no selectivity for Zn^2+^ over Cu^2+^, a divalent ion of similar size and coordination chemistry [7]. A few years later, the same team reported a modified version of GdL^c^ which had carboxylic acid groups replacing two of the picolyl moieties (Figure 2b) [40]. This modification did alter the type of complexes formed with Zn^2+^, but again, the r_1_ slightly decreased with formation of a Zn^2+^ complex so the practical usefulness of these first two designs was rather disappointing.

DTPA and DOTA (1,4,7,10-tetraazacyclododecane-1,4,7,10-tetraacetic acid) have both been popular ligands for Gd^3+^. Contrast agents that utilize DOTA-like macrocyclic ligands have many advantages over linear DTPA-based agents, including higher thermodynamic stability, more favorable kinetic inertness [49,50], and less nephrotoxicity [51]. These characteristics make DOTA the preferred choice for in vivo use. In 2007, the first DOTA-based Zn^2+^-responsive agent was reported by Major et al. [31]. This structure, referred to as Gd-daa3, contained an iminodiacetate group attached to an extended side-chain on DO3A (Figure 2d). The idea here was that in the absence of Zn^2+^, the carboxyl groups on the iminodiacetate moiety bind to open water coordination sites on the Gd^3+^ and hinder water access. As predicted, addition of Zn^2+^ resulted in a favorable 120% increase in r_1_ from 2.3 to 5.1 mM^−1^ s^−1^ due to formation of a Zn^2+^-iminodiacetate complex. When dissolved in serum, however, the basal r_1_ values both in the absence and presence of Zn^2+^ were higher, 5.8 mM^−1^ s^−1^ in the absence of added Zn^2+^ and 7.7 mM^−1^ s^−1^ after further addition of Zn^2+^ [31]. This was likely the first indication that low molecular weight agents such as Gd-apa3 do interact with serum proteins. A modified version of Gd-daa3 containing one pyridine and one acetate arm was later introduced (Figure 2e). Based on the results of these two papers, the authors concluded that at least one appended iminoacetate group must be present to hinder water access to the Gd^3+^ ion [42]. Somewhat later, Mishra et al. reported another GdDOTA derivative that shows in increase in hydration state from zero to one following the binding of Zn^2+^ (Figure 2g), but, interestingly, this probe also responded to Ca^2+^ [44].

In 2009, Esqueda et al. [43] were first to report that Zn^2+^-sensitive agents such as these form ternary complexes between Zn^2+^ and HSA. Like several earlier agents, GdDOTA-diBPEN (Figure 2f) showed only a modest increase in r_1_ relaxivity from 5.0 to 6.0 mM^−1^ s^−1^ upon addition of Zn^2+^, but the surprising finding was that this effect was greatly amplified in the presence of HSA, with r_1_ increasing to 17.4 mM^−1^ s^−1^ after addition of Zn^2+^. Taking a hint from the earlier observations of Major et al., the authors suggested that the increase in r_1_ reflected formation of a ternary complex, which would decrease molecular rotation (τ_R_). One might expect the agent to bind weakly with HSA simply through non-specific electrostatic interactions, but binding of Zn^2+^ to one or both BPEN side-chains clearly increased the affinity of the agent for HSA [43,52]. Like the first agent reported by Hanaoka et al. (Figure 2a) [39], GdDOTA-diBPEN also has two Zn^2+^ binding units, but there was no evidence found for formation of a tetrahedral Zn^2+^ complex. Instead, as described below, GdDO3A-BPEN with one Zn^2+^ binding side-chain was later shown to work equally well [49].

A similar compound, GdDOTA-diBPYREN [45], in which BPEN was replaced by the weaker Zn^2+^ binding 3-methylpyrazolyl (BPYREN) groups (Figure 2h), was also reported. The r_1_ of this modified agent was 4.2 mM^−1^ s^−1^ in aqueous buffer alone, 8.4 mM^−1^ s^−1^ in the presence of HSA, and 5.9 mM^−1^ s^−1^ in the presence of Zn^2+^. In the presence of both HSA and Zn^2+^, however, the relaxivity increased to 15.3 mM^−1^ s^−1^, so even though the 3-pyrazolyl groups have a lower affinity for Zn^2+^, this chemical difference seemed to improve the binding interaction between the agent and HSA [45].

Given that GdDOTA-diBPEN is a bis-amide bis-acetate complex, it was anticipated that the rate of water exchange in this complex might be slower than optimal for reaching the highest r_1_ possible [53]. To test this hypothesis, two other derivatives were prepared where either an acetate or an amide side-chain had extra CH_2_ groups inserted. This structural difference had been reported to increase the water exchange rate substantially and, indeed, this modification resulted in two new GdDOTA-diBPEN derivatives that displayed r_1_ values of 47.6 and 50.1 mM^−1^ s^−1^ when measured in the presence of both HSA and Zn^2+^ [54]. This remarkable 3-fold increase in r_1_, however, did not fully translate into a 3-fold increase in sensitivity for detection of Zn^2+^ release from the mouse pancreas in vivo [45,52,54]. These findings will be discussed in the next section. In a subsequent publication, GdDOTA-diBPEN was further simplified by removing one of the BPEN side chains to test whether both Zn^2+^ binding sites were indeed necessary or rather, one would work equally well. Two mono-side-chain derivatives, one with a nM binding affinity for Zn^2+^ (GdL1) (Figure 2k) and one with a μM binding affinity for Zn^2+^ (GdL2), were compared for detection of glucose-stimulated zinc-secretion (GSZS) from the mouse pancreas in vivo [48]. Although the r_1_ relaxivity of these two agents was not as high compared to the water exchange rate optimized GdDOTA-diBPEN derivative described above [54], both worked in vivo, and in fact, the lower Zn^2+^ affinity agent was considered slightly more sensitive for detection of focally intense “hot spots” in the tail of the pancreas after administration of glucose to stimulate insulin secretion. A binding model was presented to show that GdL2 has a larger dynamic range because the background signal arising from this agent binding to physiological levels of Zn^2+^ is lower initially, so that when Zn^2+^ levels rise after GSZS, the delta increase in MRI intensity is easier to detect [48]. In rodents, the pancreas is not a solid organ and can be difficult to distinguish from the surrounding tissues [55]; so, in this study, the authors implanted a MR-compatible window to fix the location of the pancreas and reduce abdominal motion due to peristalsis. This modification resulted in a surprising result in that local “hot spots” were detected in the tail of the mouse pancreas after injection of either GdL1 or GdL2 plus glucose [48]. Similar “hot spots” have also been detected in the monkey pancreas during continuous infusion of an earlier version of GdDOTA-diBPEN [56]. Although the origin of these enhanced image regions is still under investigation, they appear to be consistent with the location of individual islets or clumps of islets near highly vascularized regions of pancreatic tissue. It was suggested that these hot spots may reflect “first responder” islets, islets that deplete their entire insulin content in response to an increase in plasma glucose, while other islets release insulin more gradually over longer periods of time [57]. This implanted window technology combined with bimodal fluorescence/MRI zinc-sensitive agent, such as that reported by Stasiuk et al. [47], could be quite useful in proving this distinction. If further studies prove this to be true, this offers the possibility of using this imaging technology to examine local insulin secretion in the pancreas after treatment with new drugs under development to treat type 2 diabetes.

A few bimodal MRI and fluorescence Zn^2+^-sensitive probes have also been reported. The luminescence properties of these sensors are valuable because they can confirm any in vivo observations made by MRI, perhaps after tissues are removed. Hanaoka et al. [41] reported a lanthanide-based DTPA-quinoline DPA conjugate, using Gd^3+^ for a dual-purpose MR and fluorescent probe (Figure 2c), and europium (Eu^3+^) for a luminescent sensor. While the fluorescent sensor showed promise, the relaxivity of the bimodal sensor did not show a significant change in the presence of Zn^2+^ [41]. Also, Luo et al. developed a DOTA-based bimodal probe with a quinoline derivative arm for Zn^2+^ binding [46]. However, the two bimodal probes introduced by Hanaoka et al. [41] and Luo et al. [46] did not show ratiometric fluorescence changes in response to Zn^2+^ binding [47]. Isaac et al. also introduced a DOTA monoamide complex with Gd^3+^ or Eu^3+^ for a dual MRI and fluorescence imaging purposes [58]. Another promising bimodal probe, introduced by Dong et al., showed a 130% increase in T_1_ and bright green emission in fluorescence in response to the presence of Zn^2+^ [59].

Gd.1 (Figure 2j) consisting of a DO3A macrocycle and an amidoquinoline (AQA) Zn^2+^-sensing motif had a relaxivity of 4.2 mM^−1^ s^−1^ that increased to 6.6 mM^−1^ s^−1^ upon binding to Zn^2+^. The DO3A-AQA conjugate could bind to either Gd^3+^ or Eu^3+^, creating bimodal MRI/fluorescent Zn^2+^ sensors. Upon binding with Zn^2+^, both lanthanide probes showed ratiometric fluorescent changes with a Stokes shift from 410 to 500 nm. Albeit weakly, both sensors were able to detect Zn^2+^ in pancreatic β-cells of mice [47].

Malikidogo et al. introduced a different bimodal zinc sensor (Figure 2l) that can be used in both MRI and single-photon emission computed tomography (SPECT) [60]. Although a combination of MRI and either positron emission tomography (PET) or SPECT had been used before for pH mapping, this novel lanthanide-based agent was designed to quantify the concentration of Gd^3+^ in tissues using the SPECT properties of ^165^ER to obtain quantitative measurements of Zn^2+^ [60].

Other zinc-sensing conjugates for Gd^3+^ that should be mentioned are pyridine-based ligands. Although these sensors worked in vitro, the authors who introduced these agents concluded that they were not suitable candidates for in vivo use because of their low stability and small, non-monotonic response to Zn^2+^ [61]. In addition to Zn^2+^-responsive Gd^3+^-based MR contrast agents, other approaches have been suggested for detecting Zn^2+^ in tissues. For example, Bony et al. reported a D-glucuronic acid-coated ultrasmall Gd_2_O_3_ nanoparticle for Zn^2+^ sensing. When Zn^2+^ was added to the solution containing the probe, both r_1_ and r_2_ increased. The relaxivities changed due to an increase in water associated with the gadolinium oxide nanoparticle and an increase in its rotational tumbling time [62].

Other methods for detecting Zn^2+^ include the use of 1,2-bis(2-amino-5,6-difluorophenoxy)ethane-N,N,N′,N′-tetraacetic acid (TF-BAPTA), a Zn^2+^ binding probe combined with chemical exchange saturation transfer (CEST) MRI methods to differentiate malignant and healthy prostate cells by detection of glucose-stimulated zinc-secretion from the healthy cells [63]. Another novel approach was the use of Fe3O4@Polydopamine@DNA nanoprobes to detect the toehold-mediated strand displacement reaction (TSDR), by utilizing magnetic guidance in order to introduce the nanoprobes into the cells [64]. In TSDR, a partially double-stranded DNA is formed by a stepwise branch transfer that hybridizes a dangling region called the toehold and a single-stranded DNA [65,66]. By customizing the base sequence order and length of the toehold, TSDR enables a programmable DNA hybridization system [67]. It was suggested that such nanoparticles could recognize malignant and normal cells based on the detection of intracellular Zn^2+^ levels [64]. In yet another approach, Yu et al. used a thulium-based agent with a DO3A core, a pyridine chelate, and a perfluoro-tert-butyl tag which was only visible by ^19^F NMR in the presence of Zn^2+^. The authors concluded that this signal results from slower chemical exchange upon Zn^2+^ binding, which leads to a rigid conformation of the Zn^2+^ chelator [68]. Other thulium-based sensors have also been reported [69]. Finally, manganese-zinc ferrite nanoparticles [70,71] and manganese-porphyrin bimodal agents [72] are among other types of sensors that are also being explored. The interest in non-Gd^3+^-based agents stems from the studies that have reported adverse effects for Gd^3+^-based MRI agents, most notably, nephrogenic systemic fibrosis (NFS) in patients with severe renal insufficiency [73]. In addition to NSF, other complications such as allergic reactions [74,75] and Gd deposition in the brain [76] have also been reported.

#### 2.3.2. In Vivo Detection of Zinc with MRI

The detection of zinc by MRI has numerous potential clinical applications. The most likely targets would include disorders of the pancreas, prostate, and brain, the three organs with the most abundant zinc. MR detection of zinc in the brain is challenging, as the delivery of these types of MRI contrast agents across the blood–brain barrier is largely restricted. A Mn-porphyrin derivative has been reported that has a r_1_ relaxivity of 8.7 mM^−1^ s^−1^ in the absence of Zn^2+^, which decreases to 6.65 mM^−1^ s^−1^ upon formation of the zinc complex. This agent was directly injected into mouse brains and reportedly highlighted only zinc-rich regions of the hippocampus [77]. Although this type of agent was not shown to respond to changes on Zn^2+^ levels in the brain, it does represent a different type of MR-responsive Zn^2+^ sensor if it indeed accumulates only in Zn^2+^-rich regions of the brain. This approach could provide exciting new insights into Zn^2+^ homeostasis in various mouse models of brain abnormalities.

**Figure 3:**
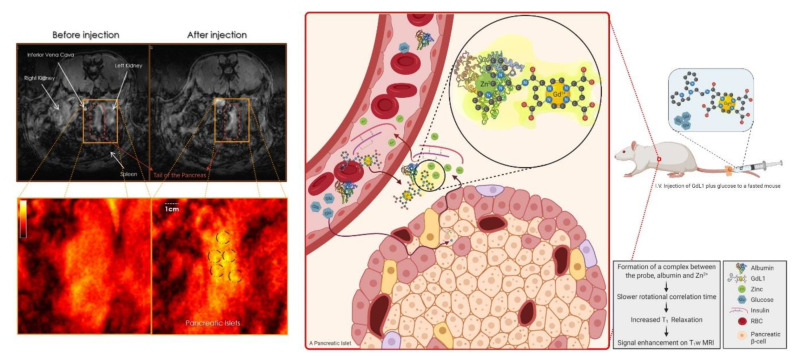
The schematic on the right shows the in vivo mechanism of action of GdL1, a zinc-responsive gadolinium-based magnetic resonance imaging (MRI) contrast agent. Upon intravenous injection of GdL1 with glucose to a fasted rat, glucose stimulates the pancreatic β-cells to secrete insulin and the co-stored zinc ions. In the interstitial tissue, GdL1 binds to zinc and albumin, forming a ternary complex that results in a slower rotational correlation time, a decrease in T_1_, and signal enhancement in T_1_-weighted MR images. The MR scans on the left are obtained before and after injection of GdL1 and glucose to a fasted rat, showing pancreatic enhancement after the injection. The dashed black circles show the hotspots that are thought to be the fast-acting islets known as “first responder” islets.

Among the many zinc-responsive MR contrast agents summarized above, the most successful in vivo applications to date involve physiologic and pathologic studies of the pancreas and prostate. GdDOTA-diBPEN was the first zinc-responsive MR contrast agent applied in mice in vivo [52]. In those experiments, fasted mice were given a low dose of GdDOTA-diBPEN ± glucose to stimulate insulin secretion from the pancreas. Difference images before and after injection of the agent plus glucose showed significant image enhancement in abdominal regions consistent with the head and tail of the pancreas, presumably reflecting insulin and Zn^2+^ secretions from those regions. MR images of the pancreas were not enhanced by the agent in the absence of added glucose nor were images enhanced in streptozotocin-treated mice with or without added glucose. Images of mice fed a high-fat (60%) diet over a 12-week period and subjected to this same imaging protocol showed a larger volume of contrast-enhanced pancreatic tissue, consistent with the expansion of pancreatic β-cell mass during progression toward type 2 diabetes [52]. In subsequent experiments, the one-armed versions, GdL1 and GdL2, were also shown to work in vivo [48] and, for the first time, focal “hot spots” reflecting regions of greater Zn^2+^ release were detected in the mouse pancreas. Figure 3 shows schematically how GdL1 is thought to work in vivo. The crucial role of Zn^2+^ in crystallization and storage of insulin in β-cells is well-established, and the extracellular concentration of Zn^2+^ around β-cells is known to increase by about 10-fold during active secretion of insulin [78,79]. It should be noted that all Gd^3+^-based contrast agents relax water protons to some extent (concentration-dependent) so that images of the pancreas are enhanced even before injection of glucose due to a low concentration of extracellular Zn^2+^ near β-cells even before additional Zn^2+^ is released by stimulation of insulin secretion by glucose. Thus, it is important to choose appropriate controls and minimize the background signal in vivo [80]. Although r_1_ can be further improved by optimizing the rate of water exchange in these Gd^3+^-based agents [54], it was interesting to find that a 10-fold increase in r_1_ as measured in vitro did not translate to a 10-fold improvement image enhancement in vivo. This likely reflects the greater complexity of the molecular environment of these agents in vivo compared to simple aqueous solutions and presents a strong argument for testing all new agents in vivo.

In 2015, a Zn^2+^-sensitive fluorescence agent was injected directly into the mouse prostate to demonstrate the changes that occur in total tissue Zn^2+^ content during the progression of prostate cancer in TRAMP (transgenic adenocarcinoma mouse prostate) mice [81]. It has long been known that Zn^2+^ transporters are downregulated as prostate epithelial cells turn malignant and consequently, the total zinc content in those cells drops dramatically in prostate cancer [82,83]. About one year later, Clavijo-Jordan et al. reported that Zn^2+^ is also released from prostate epithelial cells in response to an increase in plasma glucose levels and this secretion can be detected by MRI using the same Zn^2+^-sensitive agents used previously in studies of the pancreas [84]. This surprising observation offered the possibility of detecting prostate cancer by loss of an enhanced water signal after an injection of glucose. This indeed was demonstrated in the TRAMP mouse model where the prostate of young mice were uniformly enhanced but then began to show dark spots on a bright background over time as tumors developed in the prostate [84,85]. In a second study [85], prostate tissue was removed after the MRI exam and prepared for synchrotron radiation X-ray fluorescence (SR-XRF) to image the distribution of zinc and gadolinium in those tissues. Those images showed that the lateral lobe of the mouse prostate uniquely accumulates higher amounts of Zn^2+^, 1.06 ± 0.08 mM, while the Zn^2+^ concentration was lower, averaging 0.370 ± 0.001 mM in regions of malignant neoplasia. The SR-XRF data also showed that glucose promotes the movement of Zn^2+^ pools (∼1 mM) from the glandular lumen of the lateral lobe into the stromal/smooth muscle surrounding the glands. More recently, GdL1 was used to detect glucose-stimulated Zn^2+^-secretion in older dogs with clinically diagnosed benign prosthetic hypertrophy (BPH) [86]. This observation is important because in man, differentiation of prostate cancer from BPH tissues on MRI scans can be challenging, so translation of this technology to the clinic could be diagnostically important. In an effort to identify the connection between high glucose and Zn^2+^ from prostate cells, S961 (an insulin receptor blocker [87]) and WZB-117 (a GLUT-1,4 inhibitor [88]) were administered to rats to test whether these pharmacological agents impact Zn^2+^ secretion from the prostate. While blocking GLUT-1,4 transporters did eliminate Zn^2+^ secretion as expected, inhibition of insulin receptors did not affect the process. Interestingly, pyruvate has been shown to stimulate Zn^2+^ secretion from the rat prostate, much like glucose. This observation suggests that there may be a signal downstream of glycolysis that initiates secretion of Zn^2+^ from prostate epithelial cells [89].

### 2.4. Clinical Indications of Zinc Detection Using Magnetic Resonance Imaging

Although zinc is considered a trace mineral, it plays critical roles in various types of cellular processes and intercellular signaling [8], and it can be anticipated that an imbalance in zinc homeostasis can lead to disruptions in multiple processes, presenting as pathologies such as autoimmunity [16] or malignancy [19]. Tracking in vivo metabolism of zinc is not only interesting for studying multiple syndrome-like pathologies of zinc deficiency [90], but also for better understanding the underlying pathogenesis of many disorders that have been linked with zinc homeostasis, including Alzheimer’s disease [15] or prostate cancer [13]. Two of the most common disorders of the prostate are BPH and prostate cancer.

The prostate is an exocrine gland, producing a fluid rich in glucose, fructose, citric acid, zinc, and prostatic-specific antigen (PSA) [91]. The prostate has very high concentrations of Zn^2+^ and this is considered essential for partial inhibition of the TCA cycle at the level of aconitase [92] to produce excess citrate from carbohydrates [14]. BPH is one of the most common benign conditions in men, and as the name indicates, the condition is due to excess growth of prostate tissue. More than 85% of men older than 80 years of age have this condition [93]. Although the driving mechanism for this excessive growth is not fully understood, it is postulated that zinc metabolism is linked to the process [94,95]. Thus, MRI with zinc-responsive contrast agents can potentially aid in studying the pathogenesis of BPH.

Another prevalent disorder of the prostate is prostate cancer, which is the most common non-dermatologic cancer in men. A close link between Zn^2+^ homeostasis and malignant proliferation of the prostatic tissue has been discovered [94,95,96]. Although it is not yet clear if the low concentration of Zn^2+^ in the prostate is the cause or the effect of malignancy, it can be anticipated that in vivo evaluation of the distribution of Zn^2+^ in prostate cells during different stages of prostate cancer would provide additional insights into the pathogenesis of prostate cancer and its risk factors. In addition, serum PSA level has been used traditionally as a screening tool for prostate cancer, yet this test is neither sensitive nor specific [97]. Transrectal ultrasound (TRUS) has been the primary imaging modality used for prostate cancer, but it also has mediocre specificity and suboptimal sensitivity [98]. MRI, however, has been gaining popularity in the evaluation of prostate cancer, with or without biopsies [99,100,101]. The combination of MRI’s increasing role in the diagnosis of prostate cancer and the unquestionable part that zinc plays in the pathogenesis of prostate cancer can lead to an improvement in conventional MRI’s sensitivity and specificity for the diagnosis of prostate cancer. Results from the previously discussed in vivo experiments done in TRAMP mice, a model for prostate cancer, are evidence supporting this theory.

On the pancreatic front, there are two common disorders in which MR detection of Zn^2+^ secretion may help inform: diabetes mellitus and chronic pancreatitis. It is very well known that Zn^2+^ is a crucial factor in the crystallization and storage of insulin [78,79], showing a broader impact of zinc on both types of diabetes [12]. β-cell function, glucose metabolism, insulin activity, and pathogenesis of diabetes are directly linked to zinc homeostasis [12,102,103]. With convenient and widely available methods for checking blood glucose levels, clinicians do not need a MR scan to diagnose diabetes, yet there are many unanswered questions about diabetes. Many of the available antidiabetic drugs do not have a fully understood mechanism of action [104]. Since MR detection of zinc secretion from the β-cells directly reflects cell function, visualization of the progression of diabetes, or its regression following proper treatment, could open doors to a new perspective. β-cell function is also abnormal in chronic pancreatitis. In the late stages of the disease, islets are digested with the leaking pancreatic enzymes, leading to insulin-dependent diabetes [105]. During the early stages of chronic pancreatitis, when acute pancreatitis is transitioning into the chronic phase, the body can maintain normal glucose homeostasis, as the remaining pancreatic islets compensate for those destroyed [106]. Although this cannot be diagnosed via currently available diagnostic methods, detection of the β-cell function using MRI can potentially detect progression from acute to chronic pancreatitis. Early diagnosis of progression toward chronic pancreatitis could have a considerable impact on public health, as not only is it the most important risk factor for pancreatic cancer, but it annually costs our healthcare system more than $2 billion [106,107].

Many other conditions have been linked to Zn^2+^ homeostasis. It has been shown that male infertility is correlated with low Zn^2+^ levels in the ejaculate, but it is not fully understood whether this is the cause or an effect of infertility [108]. Zinc imbalance is also either a cause or a consequence of Alzheimer’s disease. In this disorder, amyloid plaques bind avidly with Zn^2+^ so the use of MRI to detect the presence of areas of high Zn^2+^ levels in the brain could potentially lead to early detection of Alzheimer’s disease [15,109]. Moreover, relatively high concentrations of Zn^2+^ have been reported in other tissues, including bone, skeletal muscle, liver, testicles, and the eye [10], making them potential candidates for detection of Zn^2+^ by MRI in the future.

## Figures and Tables

**Table 1:** Characteristics of some zinc-responsive Gd^3+^-based magnetic resonance (MR) contrast agents.

Contrast Agent	First Author (Year)	B_0_, Temperature	r_1_ (mM^−1^s^−1^)		K_D Zn_	Molecular Structure
			Without the Trigger ^Ϯ^	With the Trigger ^Ϯ^		
GdL^c^	Hanaoka (2001) [39]	300 MHz, 25 °C	6.06	3.98	-	Figure 2a
(GdL^a^)^2−^	Hanaoka (2002) [40]	300 MHz, 25 °C	4.8	3.4	-	Figure 2b
Gd-7	Hanaoka (2004) [41]	20 MHz, 25 °C	6.05	5.81	59 nM	Figure 2c
Gd-daa3	Major (2007) [31]	60 MHz, 37 °C	2.3	5.1	240 μM	Figure 2d
Gd-apa3	Major (2008) [42]	60 MHz, 37 °C	3.4	6.9	-	Figure 2e
GdDOTA-diBPEN	Esqueda (2009) [43]	23 MHz, 37 °C	5.0	17.4	33.6 nM	Figure 2f
Gd.L^l^	Mishra (2011) [44]	60 MHz, 37 °C	3.7	6.3	126 μM	Figure 2g
GdDOTA-diBPYREN	De Leon (2012) [45]	23 MHz, 37 °C	4.2	15.3	379 μM	Figure 2h
Gd.L	Luo (2012) [46]	23 MHz, 25 °C	3.8	5.9	-	Figure 2i
Gd.l	Stasiuk (2015) [47]	400 MHz, 37 °C	4.2	6.6	22 μM	Figure 2j
GdL1	Martins (2018) [48]	23 MHz, 37 °C	4.8	17.8	118 nM	Figure 2k

^Ϯ^ Zn^2+^ with or without human serum albumin (HSA).
